# Correlates of minimum dietary diversity among pregnant women on antenatal care follow up at public health facility in Puntland, Somalia

**DOI:** 10.1038/s41598-023-48983-9

**Published:** 2023-12-11

**Authors:** Feiruza Mohammed, Najma Abdirizak, Abdulfetah Jibril, Abdu Oumer

**Affiliations:** 1 Faculty of Nutrition, University of Health Science, Bossaso, Puntland Somalia; 2 University of Health Science, Bossaso, Puntland Somalia; 3 United Nations High Commissioner for Refugees, Bossaso, Puntland Somalia; 4 Department of Public Health, College of Medicine and Health Science, Dire Dawa University, Dire Dawa, Ethiopia

**Keywords:** Health care, Epidemiology, Risk factors

## Abstract

In Somalia, where a poorly diversified diet is leading to adverse pregnancy and neonatal outcomes, there is a significant dearth of evidence that needs to be studied. Hence, this study was to identify factors associated with minimum dietary diversity among pregnant women in Somalia. A facility-based survey was conducted among 361 pregnant women attending antenatal care (ANC) using a structured questionnaire. Dietary diversity was measured using consumption of 10-food groups. Bivariable and multivariable binary logistic regression analyses were used, along with odds ratios and 95% confidence intervals. About 48.2% (42.9–53.5) of women had an inadequately diversified diet. The risk of having an inadequately diversified diet was higher among rural residents (AOR = 1.20; 0.30–4.75), multigravida (AOR = 2.85; 1.43–5.68), young women (AOR = 2.15; 0.82–5.61), extended families (AOR = 1.19; 0.68–2.10), with infrequent ANC visits (AOR = 4.12; 2.06–8.27), fewer frequent meals (AOR = 1.84; 1.09–3.10) and from food-insecure households (AOR = 3.84; 2.28–6.49) as compared to their counterparts. Consumption of poorly diversified diet was prevalent and associated with dietary diversity was prevalent among women which could be strongly predicted by parity, ante-natal care and food security, which needs to be targeted for interventions.

## Introduction

Protecting women's nutrition at all stages of their lives is critical, especially during pregnancy and breastfeeding, when nutritional vulnerability is greatest^1^. Optimal maternal nutrition plays a crucial role in promoting healthy pregnancy outcomes and ensuring the well-being of both the mother and the developing fetus s^2^. Hence, diversified dietary consumption is one of the most cost-effective and sustainable strategies to prevent sequels of maternal malnutrition^3^. Considering this, the Food and Agricultural organization (FAO) recommended a validated tool, the Minimum Dietary Diversity for Women of Reproductive Age (MDD-W), for assessing diet quality for women. Hence, adequately diversified diet is as having at least five food groups over the past day^4^, which is highly predictive for a higher probability of nutrient adequacy for eleven micronutrients^5^.

Different forms of malnutrition are prevalent in Africa^6^. In Somalia, vitamin A, iodine, zinc, and folate deficiencies are potentially prevalent, which could increase the risk for adverse pregnancy outcomes^7,8^. Further studies in Ethiopia, Kenya, Nigeria, Somalia, and South Africa have indicated inadequate micronutrient intake of iron, vitamin A, iodine, folate, and zinc in pregnant women^7^. Based on a national survey, 47.4% of pregnant women had anemia, and 11.3% were underweight. More specifically, in Puntland, 62.6% of pregnant women had anemia. Less than one-third (30.9%) of pregnant women had an adequately diversified diet. The survey further indicated that the primary cause of micronutrient malnutrition is low micronutrient intake^9^**.**

Poor nutrient intake is one of the modifiable risk factors for maternal malnutrition, childhood stunting, and associated complications^2^. It is also evident that malnutrition of any form is unacceptable in many parts of the world, especially in Sub-Saharan Africa^10^, where micronutrient deficiencies are still a major public health concern impacting maternal health and survival and their children^2^. It can permanently affect the physiological development of the fetus, increase the risk of intrauterine growth restriction, low birth weight, preterm delivery, maternal morbidity and mortality^2^, and adulthood chronic disease risks^11^. For instance, adequate nutrient intake could reduce low birth weight by 19%, small-for-gestational-age births by 8%, preterm birth by 16%, and infant mortality by 15%^2^. Still, there is a prevailing malnutrition in Somalia, where 48.7% of women are anemic, 10.3% are stunted, 27.5% are thin, 16.6% are overweight, and 5.9% are obese, mainly due to poor dietary habits^9^.

Globally, diversified diet consumption is very limited in fruits, vegetables, dairy, fish, and meat varieties. This could lead to inadequate intake of key essential nutrients, iodine, iron, folate, calcium, and zinc, which contributed to 7% of the global disease burden due to maternal deaths, anemia, pre-eclampsia, and hemorrhage^1,10,12,13^. It had been leading to low birth weight, stillbirth, premature rupture of membranes, intrauterine growth restriction, and intrauterine fetal death^3,14^. Moreover, it is a modifiable risk factor of public health importance in the effort to prevent adverse birth outcomes, particularly among developing/low-income populations^2^. To improve it, pregnant women's dietary diversification status needs to be sustainably improved by addressing information gaps and removing barriers to adherence. Micronutrient intake should be increased through increased dietary diversity^7,15^. Hence, it appears to promote nutrient adequacy in women and lower the risk of a negative pregnancy outcome^16–18^. Moreover, diversifying one's diet is an appealing strategy for addressing nutrient deficiencies^19^. Pregnant women who eat a well-balanced diet have fewer complications during pregnancy and labor, and they are more likely to have babies who are born alive, normal, and healthy^4,20^.

As far as our knowledge is concerned, there is a lack of evidence on the coverage of minimum dietary diversity and its associated factors among pregnant women in the study area, where maternal malnutrition was prevalent. Hence, it is crucial to build context-specific evidence on diversified diet consumption and potential contributing factors for evidence-based intervention in the Somali context. Therefore, the current study was to explore the coverage of minimum dietary diversity and its associated factors among pregnant women in Puntland, and Somalia.

## Results

### Basic characteristics of study subjects

A total of 361 pregnant women were recruited in this study with 100% response rate. The mean age of participants was 26.0 years (± 5.74), and half (50.4%) of women were aged 25–34 years. The majority (91.7%) of respondents were married. In addition, more than half (53.7%) pregnant women and 32.7% of husbands were illiterates, respectively. Regarding the occupation, (74.2%) of pregnant women were unemployed while, 65% their husbands were employed. Around (62.6%) of pregnant women had a family size below 5 people and majorities (97%) were residing in urban with (88.9%) obtain their food from the market. In terms of wealth, 19.9% of participants were in the wealthiest, while 19.9% were in the poorest socioeconomic class (Table 1).

**Table 1:** Socio-demographic and socio-economic status of pregnant women attending ANC clinic at public health facilities in Bossaso City, Puntland, Somalia, May 2023.

	Variables	Frequency	Percentage
Age category in years	< 25	144	39.9
	25–34	182	50.4
	> 34	35	9.7
Marital status	Married	331	91.7
	Single	2	0.6
	Widowed	7	1.9
	Divorced	20	5.5
Maternal education status	Unable to read and write	194	53.7
	Read and write	65	18
	Elementary school	29	8
	High school	55	15.2
	College and above	18	5
Head of household education status	Unable to read and write	118	32.7
	Read and write	91	25.2
	Elementary school	29	8.0
	High school	50	13.9
	College and above	73	20.2
Residence	Urban	350	97.0
	Rural	11	3.0
The main source of food	Market	321	88.9
	NGO/support	8	2.2
	Farm/garden	7	1.9
	Relatives/friends	7	1.9
	Other/specify	18	5.0
Employment status	Employed	93	25.8
	Unemployed	268	74.2
Husband’s employment (n = 334)	Employed	217	65.0
	Unemployed	118	35
Wealth status	Poorest	22	6.1
	Poorer	122	33.8
	Middle	71	19.7
	Wealthier	74	20.5
	Wealthiest	72	19.9

### Obstetric and maternal health status of pregnant women

Regarding the obstetric and maternal health status of pregnant women, the majority (81.7%) were multigravida (got more than one pregnancy), 44.3% of them were in the second trimester, and nearly half (47.9%) of them had less than four ANC visits. More than half (52.1%) of the participants were unaware of the significance of food diversity, while 51.8% had avoided any foods during their current pregnancy. And 33% reported avoiding food mainly due to personal preferences. In terms of meal frequency, the majority of pregnant women (70.9%) ate at least three times per day, and 59.6% were ill in the first two weeks of the data collection period, with 23.8% were anemic (Table 2).

**Table 2:** Obstetric and maternal health status of pregnant women attending ANC clinic at public health facilities in Bossaso City, Puntland, Somalia, May 2023.

Variables	Options	Frequency	Percentage
Parity	Primigravida	66	18.3
	Multigravida	295	81.7
ANC visit	1st visit	112	31.0
	2–3 visits	173	47.9
	≥ 4th visits	76	21.1
Trimester	First trimester	73	20.2
	Second trimester	160	44.3
	Third trimester	128	35.5
Reason for food avoidance	Personal dislike	119	33.0
	Cultural food taboo	5	1.4
	Will make baby big and labour difficult	27	7.5
	Others^a^	36	10.0
Meal frequency	< 3 meal	105	29.1
	≥ 3 meal	256	70.9
Illness in last 15 days	No	146	40.4
	Yes	215	59.6
Type of illness	No illness	146	40.4
	Anemia	86	23.8
	Malaria	15	4.2
	Typhoid	16	4.4
	Gastritis	37	10.2
	Urinary tract infection	44	12.2
	Others^b^	17	4.8
Information about the importance of food diversity	No	188	52.1
	Yes	173	47.9
Avoid any foods during current pregnancy	No	174	48.2
	Yes	187	51.8
Reasons for avoiding food	Food not avoided	174	48.2
	Personal dislike	119	33.0
	Cultural taboo	5	1.4
	Will make baby big and labour difficult	27	7.5
	Illness (gastritis)	24	6.6
	Others^c^	12	3.3

^a^Refers to peer influence, lack of access and lack of appetite; ^b^refers to vaginal infections and back pain; and ^c^refers to women who avoid due to food allergy.

### Magnitude of household food insecurity

**Figure 1:**
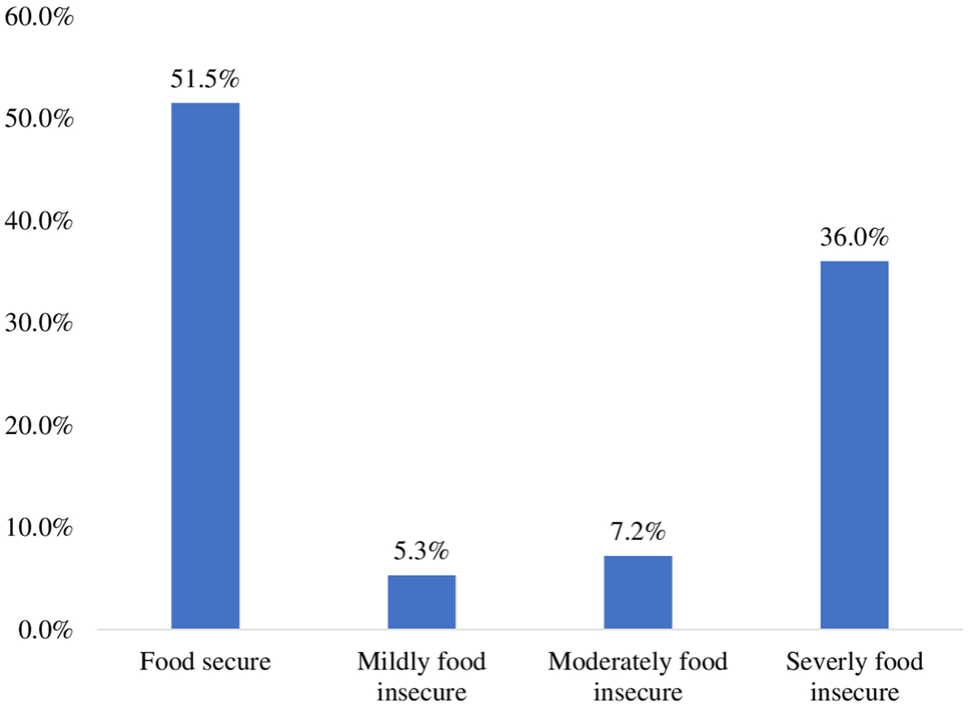
Household Food Security Status of Pregnant Women attending ANC clinic at public health facilities in Bossaso City, Puntland, Somalia, May, 2023.

More than half (51.5%) of women were from food-secure households, 5.3% were mildly food-insecure, and 7.2% were moderately food-insecure households. Unfortunately, 36.0% of them were from severely food insecure households, affecting their future livelihood (Fig. 1).

### Coverage of inadequately diversified diet among pregnant women

The most commonly consumed food group by many pregnant women (92.0%) in the previous 24 h was grains, white roots and tubers, and plantains. The main grains consumed were rice, sorghum, and wheat "anjero," bread, pasta, macaroni, and porridge, which were considered the staple foods in the area, followed by other vegetables (78.9%) and dairy (65.7%) in the form of milk. Furthermore, 61.8% of participants ate meat, 50.7% fed on dark green leafy vegetables from spinach and cabbage, 44.9% and 27.7% consumed plant-based proteins from the pulses group (beans, peas, and lentils) and nuts and seeds, respectively, and 47.1% fed on other vitamin A-rich vegetables and fruit and 41.0% on eggs. The mean minimum dietary diversity score was 4.93 (2.56 standard deviation), and more than half (51.8%: 95% CI 46.5, 57.1) of pregnant women had adequate dietary diversity for women (MDD-W), while the remaining 48.2% (95% CI 42.9, 53.5) had inadequately diversified diet (Table 3).

**Table 3:** Food consumption and minimum dietary diversity among pregnant women attending ANC clinic at public health facilities in Bossaso City, Puntland, Somalia, May, 2023.

Variables	Frequency	Percentage
Food groups		
Grains, white roots and tubers, and plantains	332	92.0
Pulses	162	44.9
Nuts and seeds	100	27.7
Dairy	237	65.7
Meat, poultry and fish	223	61.8
Eggs	148	41.0
Dark green leafy vegetables	183	50.7
Other vitamin A rich vegetables and fruit	170	47.1
Other vegetables	285	78.9
Other fruits	56	15.5
Minimum dietary diversity		
Mean MDD-W score	4.93(± 2.56 SD)	
Coverage of MDD with 95% CI	174:48.2: 95% CI (42.9, 53.5)	

### Factors associated with minimum dietary diversity

Bivariable binary logistic regression was used to evaluate the association between risk factors and diversified dietary consumption. Maternal education, head of household education, wealth index, parity, ANC visits, meal frequency, and HFIAS were important factors associated with MDD-W. Multigravida mothers were associated with a 1.96-time increased susceptibility to a less diversified diet (COR = 1.96; 95% CI 1.13, 3.42). Household food insecurity (COR = 4.62; 95% CI 2.84–7.49) and less frequent meal consumption (COR = 2.19; 95% CI 1.38–3.49) were significantly associated with an inadequately diversified diet. Those who had fewer visits (COR = 5.45; 2.87–10.37) had less frequent diet consumption as compared to those who had at least four follow-ups. Diversified diet consumption is associated with lower socioeconomic class (COR = 2.91; 95% CI 1.09–7.77) and large family size (COR = 1.26; 95% CI 0.82–1.94) (Table 4).

**Table 4:** Determinants of inadequately diversified dietary consumption among pregnant women attending ANC clinic at public health facilities in Bossaso City, Puntland, Somalia.

Variables		Minimum dietary diversity-women (MDD-W)		COR (95%CI)	AOR (95%CI)	P-value
		Have inadequate MDD-W	Have adequate MDD-W			
Age group in years	< 25	70	74	1.60 (0.75–3.42)	2.15 (0.82–5.61)	1 0.119
	25–34	91	91	1.69 (0.80–3.56)	2.23 (0.93–5.34)	0.071
	> 34	13	22	1	11	
Family size	≥ 5	70	65	1.26 (0.82–1.94)	1.19 (0.68–2.10)	0.538
	< 5	104	122	1	11	
Wealth index	Poorest	12	10	2.91 (1.09–7.77)		
	Poorer	74	48	3.74 (2.01–6.99)		
	Middle	40	31	3.13 (1.57–6.26)		
	Wealthier	27	47	1.40 (0.70–2.79		
	Wealthiest	21	51	1		
Residence	Urban	168	182	1	11	
	Rural	6	5	1.30 (0.39–4.34	0.1.20 (0.30–4.75)	0.801
Maternal employment	Employed	47	46	1.13 (0.71–1.82	1	
	Unemployed	127	141	1		
Gravidity	Primi gravida	23	43	1	11	
	Multi gravida	151	144	1.96 (1.13, 3.42)	2.85 (1.43–5.68)	0.003**
ANC Visit	1st visit	74	38	5.45 (2.87–10.37)	4.12 (2.06–8.27)	0.0001**
	2nd and 3rd visit	80	93	2.40 (1.33–4.35)	2.28 (1.21–4.30)	0.011*
	≥ 4th visit	20	56	1	1	
Meal frequency	< 3 meal	65	40	2.19 (1.38, 3.49)	1.84 (1.09–3.10)	0.022*
	≥ 3 meal	109	147	11		
Food taboo	No	78	96	1		
	Yes	96	91	1.30 (0.86–1.97)		
Trimester of pregnancy	First trimester	34	39	1.20 (0.67–2.13)		
	Second trimester	86	74	1.59 (0.99–2.55)		
	Third trimester	54	74	1		
HFIAS	Food secure	12	10	11		
	Mildly food insecure	74	48	1.39 (0.53–3.62)	1.51 (0.54–4.22)	0.435
	Moderately food insecure	40	31	1.19 (0.51–2.78)	1.04 (0.43–2.54)	0.928
	Severely food insecure	27	47	4.62 (2.84–7.49)	3.84 (2.28–6.49)	0.0001**
Illness history	No	65	81	1		
	Yes	109	106	1.28 (0.84–1.95)		

Statistically significant factors associated with minimum dietary diversity at p-value < 0.05 (*) and P-value < 0.01 (**).

In multivariable logistic regression analysis, the adjusted odds ratio (AOR) for all selected variables was computed using the backward stepwise regression method to identify more significantly associated variables. Hence, important factors with a p-value below 0.25 and other important risk factors were included in the final model. Hence, parity, ANC visits, meal frequency, age, family size, and HIFAS were significantly associated with the MDD-W. Young women (AOR = 2.15; 95% CI 0.82–5.61) and pregnant women with extended family (AOR = 1.19; 95% CI 0.68–2.10) had 2.15- and 1.19-times higher odds of having inadequately diversified dietary consumption, respectively. The risk of having an inadequately diversified diet was higher among rural residents (AOR = 1.20; 95% CI 0.30–4.75), multigravida women (AOR = 2.85; 95% CI 1.43–5.68), and those with less frequent ANC visits (AOR = 4.12; 95% CI 2.06–8.27) as compared to their counterparts. Furthermore, women with fewer frequent meals (AOR = 1.84; 95% CI 1.09–3.11) and from severely food-insecure households (AOR = 3.84; 95% CI 2.28–6.49) were associated with 1.84- and 3.84-times increased odds for inadequately diversified dietary consumption (Table 4).

## Discussion

This study was aimed at assessing the coverage and identifying factors associated with minimum dietary diversity among pregnant women attending ANC clinics at public health facilities in Somalia. The findings of this study showed that 48.2% (45.6–50.8%) of pregnant women had inadequately diversified dietary consumption. Moreover, consumption of inadequately diversified diet is mainly associated with gravidity, ANC, meal frequency and food insecurity, which could be targeted for interventions.

The result of this study showed that significantly high percentage of women had an inadequately diversified diet, Which is very consistent with studies conducted in Ethiopia (51%)^21^, and North Ghana (52%)^22^. However, the findings in this study were lower than those from Bangladesh (65%)^23^, India (73.5%)^24^, Indonesia (62.5%)^25^, and Kenya (85%)^18^. The possible explanation for this discrepancy might be due to the seasonality of diversified food availability. It could also be due to the fact that some of the studies used the old nine food groups, which might underestimate or overestimate the true magnitude. Hence, in Somalia, there is a significant prevalence of inadequately diversified diets among pregnant women, which poses serious health risks for both the expectant mothers and their unborn children^26^. The traditional Somali diet heavily relies on staples like rice, maize, and sorghum, while lacking in essential nutrients such as vitamins, minerals, and proteins^26–29^. This deficiency increases the vulnerability of pregnant women to malnutrition, anemia, and other complications during pregnancy, potentially leading to adverse birth outcomes and impaired infant development^26,27,30^.

Higher estimates were also reported from India (18%)^23^, Nepal (45%)^31^, Nigeria (30.8%)^32^, Southern Ethiopia (7.9%)^33^, and Eastern Ethiopia (43%)^34^. The possible explanation for this discrepancy in magnitude might be due to study period differences and geographical and socio-cultural differences that could affect availability and access to food. However, we can understand that with the current rising food prices, poor access to nutritious food, and lower purchasing power of foods^35^, there is a potential for prevailing micronutrient deficiencies. This has been indicated in the recent national survey^9^. These could ultimately increase the risk of adverse pregnancy outcomes like low birth weight^29^. Promoting edible backyards in urban and rural settings might improve access to a diversified diet for pregnant women^30,36^.

In the present study, parity was significantly associated with minimal dietary diversity. Compared to primigravida, multigravida pregnant women were more likely to have poor dietary diversity. This is closely linked to the food insecurity associated with expanded family sizes. Sharing food among family members, could lead to a shortage of food within the household, and women usually prioritize their children, limiting their diversified dietary consumption^37^. This finding was similar to results obtained from Nigeria^32^. Related to this, women with extended families (≥ 5) had a higher susceptibility to a less diversified diet.

The other high-risk population had less frequent ANC visits; more specifically, those with their first ANC were associated with a less diversified diet compared to those who had four or more ANC visits. This could be attributable to the role of enhanced dietary counselling during the ANC visit and the receding nausea and vomiting in the later stages of pregnancy. Previous findings in Nepal had proven that the number of ANC visits ≥  4 times was significantly positively associated with increased odds of dietary diversity^31^. Increasing ANC adherence could help enhance the knowledge of mothers and ultimately improve dietary diversity. With the current WHO-recommended ANC visits, there is potential for behavioural changes to improve the nutritional status of pregnant women.

It has been found that women with a smaller meal frequency are significantly negatively associated with dietary diversity. This is mainly determined by economic access to food, where women with infrequent meals, are usually less food secure^38^. As compared to the recommended four meals for pregnant women, having infrequent meals is usually associated with an energy deficit and an increased risk of adverse pregnancy outcomes. Other studies from Northern Ethiopia^39^ and Southern Ethiopia, Hadiya zone^33^, showed a positive association with meal frequency and dietary diversity. Similarly, socioeconomic class is associated with diversified dietary consumption. This emphasizes that the purchasing power of women from lower socioeconomic classes is limited. It would be helpful to target women from lower socioeconomic classes for food security and livelihood interventions or through targeted supplementary feeding programs. In the current era, income could play a significant role in improving dietary diversity beyond nutritional knowledge^40^.

Dietary diversity is a promising indicator for food security and is inversely correlated with HFIAS^41^. Furthermore, this study clarifies that there is a significant association between food security and dietary diversity. A strong dose–response was noticed where inadequately diversified dietary consumption increases as the degree of food insecurity advances. This is consistent with previous findings from Bangladesh^42^, Ghana^43^, where it was proven that women from households with poor food insecurity were less likely to achieve the MDD-W, and Malawi^44^ as well. It is also in line with a study conducted in Ethiopia^45^. Pregnant women with severe food insecurity had a higher risk of not achieving minimum dietary diversity compared with food-secure pregnant women. Overall, the study tried to indicate the prevailing food insecurity and poorly diversified diet among pregnant women in the study area. These proxy indicators implicate poor access to a variety of foods and food insecurity, further deteriorating maternal malnutrition.

The result of this study could be compromised by the following methodological issues: first, the seasonal availability of food might affect access to diversified food. However, the current study is conducted in the lean season, which could potentially implicate the worst-case scenario. Second, the possibility of recall bias and the potential to give biased findings could not be excluded. These might lead to an overestimation or underestimation of the study's findings. However, the study pinpointed high-risk population segments with poorly diversified diets for targeting nutrition-specific and nutrition-specific interventions in the study area.

## Conclusion and recommendations

Overall, consumption of inadequately diversified diet was prevalent among pregnant women in part of Somalia and is mainly associated with multigravidity, infrequent meals, not having regular ANC, and household food insecurity which could contribute a lot to the prevailing micronutrient deficiencies. Therefore, policymakers, program managers, healthcare workers, and stakeholders need to improve nutrition promotion and intervention programs to alleviate this issue. It could be helpful to design interventions targeted at improving effective ANC coverage and food security in the study to improve diet via building better knowledge and food access. Addressing this problem requires comprehensive interventions that focus on improving food security, promoting nutritional education, and increasing the availability of diverse and nutrient-rich foods to ensure the health and well-being of pregnant women in Somalia.

## Materials and methods

### Study area and period

This study was conducted in selected health facilities from parts of Somalia named Bossaso, Puntland in the Horn of Africa. The selected health facilities are located 11.2755° N, 49.1879° E. The study was conducted from January 10 to February 25, 2023. The nation had a total population of 12.3 million as of 2020, with 51%, 23%, and 21% residing in urban, rural, and nomadic settings, respectively. Among these, 49% were female, and 50% were women of reproductive age (15–49 years)^46^. The study was conducted at public health facilities in Bossaso City, located in the north-eastern Bari region of Puntland State and on the southern coast of the Gulf of Aden, at a distance of 1400 kms from the capital city. There are an estimated 583,218 residents, with 69% of them living in cities. More specifically, Bossaso city is expected to handle 73, 137 population as of October 22, 2023^47^. In the area, sorghum and maize are the usual staple crops cultivated, yet there is access to rice and spaghetti, especially for urban dwellers^48^.

Bossaso City comprises one general public hospital, which serves as a referral hospital and provides comprehensive emergency obstetric care, and ten health centres providing basic emergency obstetric care and antenatal care services. The eight health centers are expected to provide service to an estimated 583,218 populations, where 68.9% of the catchment population are urban residents. Hence, there are an estimated monthly flow of 327, 114,183 and 295 pregnant women on monthly basis attending these facilities in central health centre, Daryeel, Boqol Bush and Gurmad health centres, respectively.

The economic livelihood of the Somalia population is largely comprised of pastoralists and agro-pastoralists, making this livelihood extremely vulnerable to climatic hazards and food insecurity, potentially affecting food consumption.

### Study design and population

A facility-based cross-sectional study was conducted from January to June 2023 among systematically selected pregnant women attending antenatal care follow-up at selected public health facilities in Bossaso City. The study excluded pregnant women whose nutritional intake is compromised by mental illnesses such as depression or psychosis; those who become seriously ill as a result of pregnancy (hyperemesis gravidarum); and repeated visits during the study period. The result will be generalizable to all pregnant women on ANC follow-up in Bossaso City, Somalia.

### Sample size estimation and sampling procedure

The required sample size for this study was estimated using different assumptions. We employed single population formula using 10% non-response rate, 95% confidence level, prevalence of diversified dietary consumption from Somalia (69.1%)^9^. The sample size for this assumption became 361. Considering the second objective, the minimum sample required was estimated using StatCalc module of EPiInfo at 5% tolerable margin of error, 80% based on previous studies^49,50^. However, the sample size required was small where we took the larger sample for the first objective 361 considering non-responses.

A simple random selection was employed to select four public health facilities in Bossaso City, followed by stratified random sampling with proportional allocation based on the total number of pregnant women visiting the 4th ANC within one month from each selected public health facility. (Central health center, n1 = 128; Daryeel health center, n1 = 45; Boqol bush health center, n1 = 72; Gurmad health center, n1 = 116). Then, systematic sampling techniques were used to select study participants from each selected public health facility, considering the sampling intervals, and the first study subject was chosen at random.

### Methods of data collection

Data were collected through a pretested interviewer-assisted interview technique using a pretested structured questionnaire structured into sections: socio-demographic and socio-economic characteristics (age, marital status, residence, education, family size, women’s occupation, household asset, and others); obstetric and maternal health characteristics; a food insecurity assessment tool; and a 10-food group detailed MDD-W tool. The MDD-W tool included cereal grains (white roots, tubers, and plantains), pulses (beans, peas, and lentils), nuts and seeds, dairy, meat (poultry and fish), eggs, dark green leafy vegetables, vitamin A-rich fruits and vegetables, other vegetables, and other fruits. The minimum dietary diversity is defined as consuming at least five food groups out of ten food groups in the past one month. The fourth part is about household food insecurity measurement tools and questionnaires. The questionnaire was prepared in English, translated into Somali by a language expert, and translated back into English for consistency checks. The study participants were approached at the MCH service unit before they started their regular ANC follow-up. Using a standardized interview protocol, the data collectors obtained verbal consent after explaining the study procedure. Fortunately, all respondents were willing to participate with null non-responses.

The MDD-W level was assessed using a FAO-validated tool where food consumption was elicited over the past 24-h period, excluding fasting and unusual periods. The actual consumption was elicited in a multiple-pass method to avoid missing food items. Respondents were asked whether they had taken any food from ten pre-defined groups. Moreover, the status of food security was assessed by using the household food insecurity access scale (HFIAS) measurement tool developed by FANTA. These two measurements are closely linked to each other in predicting nutritional status. Also, we measured food security status over the past month with the aim of capturing the usual and recent food security status of the household^41,51^.

### Operational definitions

In this study, the MDDW was categorized as adequate or inadequate depending on the dietary diversity. Hence, those who reported to consume at least five food groups in the previous day were considered as having adequately diversified diet and inadequately diversified diet instead. In addition the food security status was defined as per the FAO definition depending on the experience of each food insecurity experiences. Hence, food secure when [(Q1a = 0 or Q1a = 1) and Q2 = 0 and Q3 = 0 and Q4 = 0 and Q5 = 0 and Q6 = 0 and Q7 = 0 and Q8 = 0 and Q9 = 0]; mildly food insecure when [(Q1a = 2 or Q1a = 3 or Q2a = 1 or Q2a = 2 or Q2a = 3 or Q3a = 1 or Q4a = 1) and Q5 = 0 and Q6 = 0 and Q7 = 0 and Q8 = 0 and Q9 = 0]; moderately food insecure [(Q3a = 2 or Q3a = 3 or Q4a = 2 or Q4a = 3 or Q5a = 1 or Q5a = 2 or Q6a = 1 or Q6a = 2) and Q7 = 0 and Q8 = 0 and Q9 = 0]; and severely food insecure when [Q5a = 3 or Q6a = 3 or Q7a = 1 or Q7a = 2 or Q7a = 3 or Q8a = 1 or Q8a = 2 or Q8a = 3 or Q9a = 1 or Q9a = 2 or Q9a = 3]^41,51^.

### Data quality control

To assure the acquisition of quality data, we employed multiple data quality assurance approaches. First, we employed a pre-test on 20 pregnant women at Horseed Health Center, and we made necessary amendments to the sequencing of the questionnaire. A one-day practical training was conducted prior to data collection, focusing on the objective of the study, interview techniques, data recording, ways of obtaining consent, and how to maintain confidentiality. The significance and appropriate meanings of each question were emphasized, and participants were given clear explanations. In addition, the data collections were monitored on a daily basis by the supervisors and the investigator. Throughout the data collection period, collected data was manually checked for completeness, accuracy, and consistency prior to data entry. Moreover, we employed a quality check during data entry using check codes.

### Study variables

In this study, the dependent variable was minimum dietary diversity for pregnant women (MDD-W), measured using the standard FAO’s minimum dietary diversity for women (MDD-W) (yes/no). This was based on the minimum number of food groups consumed over the past day (five food groups out of ten)^4^,and the independent variables were socio-demographic and socioeconomic characteristics (maternal age, level of education, residence, household size, maternal occupation, household asset); obstetric and maternal health characteristics (parity, number of ANC visits, trimester, information about MDD-W, avoid any foods, meal frequency per day, illness in the last 15 days, type of illness), and household food insecurity status.

### Data management and analysis

The cross-checked hard copy data was obtained from data collectors, entered into a predefined data entry format, and exported to SPSS version 25 for processing and analysis. Descriptive analysis was used to describe, summarize, and present the data using frequency, percentage, mean, standard deviations, statistical tables, and graphs.

The HFIAS and the MDD-W items were coded properly and used to calculate the food security status and the MDD-W status according to the prespecified criteria stated elsewhere. In addition, the mean and standard deviation scores of MDD-W were calculated accordingly. Those who consumed at least five food groups out of ten diversities in the previous data was classified as having adequately diversified dietary consumption. The household wealth index measuring tool was adopted from the Puntland Health and Demographic Survey (PHDS), 2020^52^. The household wealth status was derived using principal component analysis (PCA) after exploring potential statistical assumptions. Finally, the factor scores transformed and ranked into three wealth index categories^21,31^. Food insecurity was measured using the FANTA HFIAS tool. It consists of nine occurrence questions that represent a generally increasing level of severity of food insecurity (access), and nine “frequency-of-occurrence” questions. and categorized into four levels; food secure, mildly, moderately, and severely food insecure, as per the established FAO guideline^53^.

Binary bivariable logistic regression was used to determine which variables have individual associations with MDD-W, the dependent variable. The model's goodness of fit was evaluated using Hosmer–Lemeshow’s statistic and Omnibus tests. All variables with a P-value 0.25 in the bivariable analysis were included in the final model as candidates for multivariable logistic regression in order to control all possible confounders^54,55^. In multivariable analysis, a backward stepwise regression was used to identify more significantly associated variables such as parity, number of ANC visits, meal frequency, and HFIAS. The strength of associations between variables was assessed by using crude and adjusted odds ratios at 95% confidence intervals. A p-value was declared at a p-value below 0.05.

### Ethical approval and consent to participate

Ethical approval was obtained from the Institutional Research Ethical Review Board of Dire Dawa University, as well as an official letter from the University of Health Science (UOHS) Bossaso, Somalia, which was obtained and submitted to the HC/MCH’s head. Information sheets and informed voluntary consent for the heads of each public health facility were obtained. Verified verbal informed consent was obtained from each participant. Informed consent was approved by the University’s IRB and informed voluntary consent was obtained from all pregnant women. All methods and study procedures were performed in accordance with Dire Dawa University Institutional Ethical Review Board and Helsinki Declarations.

## Data Availability

The data generated and analyzed in this study are included with the submitted manuscript.

## Abbreviations

A/COR: Adjusted/crude odds ratio
CI: Confidence interval
ANC: Antenatal care
DDS: Dietary diversity score
FSNP: Food security and nutrition policy
FANTA: Food and nutrition technical assistance
FAO: Food and Agriculture Organization of the United Nation
MCH: Maternal and child health
WHO: World Health Organization
MDD: Minimum dietary diversity
MDDS: Minimum dietary diversity score
HFIAS: Household food insecurity access scale
H/C: Health center
PDHS: Puntland Health and Demographic Survey
